# Strigolactone Can Promote or Inhibit Shoot Branching by Triggering Rapid Depletion of the Auxin Efflux Protein PIN1 from the Plasma Membrane

**DOI:** 10.1371/journal.pbio.1001474

**Published:** 2013-01-29

**Authors:** Naoki Shinohara, Catherine Taylor, Ottoline Leyser

**Affiliations:** 1 Department of Biology, University of York, Heslington, York, United Kingdom; 2 Sainsbury Laboratory, University of Cambridge, Cambridge, United Kingdom; Wageningen University, Netherlands

## Abstract

Plants continuously extend their root and shoot systems through the action of meristems at their growing tips. By regulating which meristems are active, plants adjust their body plans to suit local environmental conditions. The transport network of the phytohormone auxin has been proposed to mediate this systemic growth coordination, due to its self-organising, environmentally sensitive properties. In particular, a positive feedback mechanism termed *auxin transport canalization*, which establishes auxin flow from active shoot meristems (auxin sources) to the roots (auxin sinks), has been proposed to mediate competition between shoot meristems and to balance shoot and root growth. Here we provide strong support for this hypothesis by demonstrating that a second hormone, strigolactone, regulates growth redistribution in the shoot by rapidly modulating auxin transport. A computational model in which strigolactone action is represented as an increase in the rate of removal of the auxin export protein, PIN1, from the plasma membrane can reproduce both the auxin transport and shoot branching phenotypes observed in various mutant combinations and strigolactone treatments, including the counterintuitive ability of strigolactones either to promote or inhibit shoot branching, depending on the auxin transport status of the plant. Consistent with this predicted mode of action, strigolactone signalling was found to trigger PIN1 depletion from the plasma membrane of xylem parenchyma cells in the stem. This effect could be detected within 10 minutes of strigolactone treatment and was independent of protein synthesis but dependent on clathrin-mediated membrane trafficking. Together these results support the hypothesis that growth across the plant shoot system is balanced by competition between shoot apices for a common auxin transport path to the root and that strigolactones regulate shoot branching by modulating this competition.

## Abstract 2

Shoot branching is regulated by competition between branches to export the phytohormone auxin into the main stem. The phytohormone strigolactone balances shoot system growth by making auxin export harder to establish, thus modulating the auxin transport network.

## Author Summary

Plants can adapt their form to suit the environment in which they are growing. For example, genetically identical plants can develop as a single unbranched stem or as a highly ramified bush. This broad developmental potential is possible because the shoot system is produced continuously by growing tips, known as shoot meristems. Meristems produce the stem and leaves of a shoot, and at the base of each leaf, a new meristem is formed. This meristem can remain dormant as a small bud or activate to produce a branch. Thus, the shoot system is a community of shoot meristems, the combined activity and inactivity of which shape shoot form. Here we provide evidence that growth is balanced across the Arabidopsis shoot system by competition between the shoot meristems. This competition is likely mediated by the requirement of meristems to export the plant hormone auxin in order to activate bud outgrowth. In our model, auxin in the main stem, exported from active branches, can prevent auxin export by dormant buds, thus preventing their activation. Our findings show that a second hormone, strigolactone, increases the level of competition between branches by making auxin export harder to establish. Together, these hormones balance growth across the shoot system, adjusting it according to the environmental conditions in which a plant is growing.

## Introduction

Plants can alter their body plan to adapt to the environment in which they are growing (reviewed in Leyser 2009 [1]). This is possible because plant development is continuous, with postembryonic development being dependent on the activity of meristems. For example, the primary shoot apical meristem is laid down during embryogenesis at the apical embryonic pole, and after germination, the meristem gives rise to the adult shoot system through the production of a series of phytomers consisting of a leaf, a segment of stem, and a new shoot apical meristem, established in the axil of each leaf. These axillary meristems can remain dormant, or they can activate to produce a new shoot axis, with the same developmental potential as the primary shoot. Thus the mature shoot system can range from a solitary stem to a highly ramified bush, depending on the activity of the axillary meristems. The large number of meristems in the shoot system allows the plant to recover quickly from damage and to adjust its growth according to spatially heterogeneous environmental inputs such as unilateral shading, and to systemic inputs such as the nutrient status of the plant. Thus multiple inputs are integrated to balance growth across the shoot system.

Axillary meristem activity is controlled by a network of systemically moving endogenous signals, among which auxin plays a pivotal role. Auxin, synthesized principally in the young expanding leaves of growing shoot tips, moves rootward in the stem through the polar auxin transport stream (PATS). The direction of the PATS is determined by the polar localisation of PIN-FORMED (PIN) plasma membrane (PM) auxin efflux carriers [2],[3]. In the stem, efficient rootward auxin flow requires PIN1 [4], which is basally localised in the PM of xylem parenchyma cells [5]. Auxin in the PATS inhibits the outgrowth of axillary buds. Pharmacological inhibition of the PATS or removal of the primary shoot apex triggers outgrowth of axillary buds, and application of auxin to the decapitated stump prevents this outgrowth [6],[7]. However, direct application of auxin to axillary buds does not prevent their outgrowth [8], and apically applied auxin is not transported into the axillary buds [9], suggesting that auxin in the PATS inhibits shoot branching indirectly.

Two nonexclusive mechanisms have been proposed to account for the indirect action of auxin. Firstly, it has been proposed that auxin regulates the synthesis of one or more second messengers, which move up into the axillary buds to regulate their activity. Two classes of phytohormone, cytokinins and strigolactones, are good candidates for these signals. Cytokinins can move up the plant in the transpiration steam in the xylem. Direct application of cytokinin to axillary buds can induce outgrowth, even in an intact plant [10]. Decapitation elevates but auxin application reduces endogenous cytokinin levels in xylem sap [11] and in the stem of nodal explants [12]. Together these data suggest that auxin inhibits bud outgrowth in part by reducing systemic and local cytokinin levels, and thus cytokinin supply to buds. A similar dataset exists for strigolactones. They can also be transported up the plant in the xylem [13]. Their direct application to buds can inhibit outgrowth on intact and decapitated plants [14], and decapitation reduces but auxin application elevates the transcription of strigolactone biosynthetic genes [15],[16]. These data suggest that auxin inhibits bud activity in part by increasing systemic and local strigolactone synthesis and thus strigolactone levels in buds.

However, strigolactones only inhibit shoot branching in the presence of a competing auxin source, such that supply to a solitary bud has little or no effect and supply to an explant carrying two buds inhibits only one of the buds, which can be either the more apical or more basal bud [17],[18]. Furthermore, strigolactone addition results in a reduction in PIN1 levels in xylem parenchyma cells within 6 h, accompanied by a reduction in polar auxin transport [17]. Thus in strigolactone biosynthetic mutants, high levels of branching correlate with high levels of PIN1 and polar auxin transport and high auxin concentration in the main stem [19],[20]. These observations have led to an alternative model both for strigolactone action and for the indirect mode of inhibition of axillary bud growth by auxin in the PATS in the main stem.

This alternative model derives from considerations of the auxin transport canalization hypothesis, originally proposed to explain vascular pattern formation. The central tenet of the canalization hypothesis is positive feedback between auxin flux and auxin flux capacity [21]. Restated in terms of PIN proteins, an initial passive flux of auxin from an auxin source to an auxin sink results in the up-regulation and polarisation of PINs in the direction of the flux. This results in files of cells with high levels of PINs polarised in the direction of the sink, some of which may differentiate into vascular strands. The emergence of such files between an auxin source and sink has been directly observed [22],[23].

Given that active axillary buds are sources of auxin [23],[24], and the main stem can act as an auxin sink, by carrying auxin away to the root, auxin transport canalization can act to connect the bud to the stem, transporting auxin away from the bud apex and establishing vascular connectivity between the bud and the rest of the plant. However, high auxin levels in the main stem can prevent canalization of auxin transport out of the bud by reducing stem sink strength for auxin, limiting the initial flux of auxin out of the bud, thereby preventing escalation of the positive feedback at the heart of the canalization process [20],[23]. If auxin transport canalization out of the bud is required for bud activity, then this could explain the indirect inhibition of buds by auxin in the main stem, without the need for a second messenger relaying the auxin signal into the bud. Instead, buds and the main shoot apex compete for access to a common auxin transport pathway down to the root. Computational simulations of this model demonstrate its plausibility [20]. Moreover the model can explain the association of high branching with high PIN1, auxin transport, and auxin levels observed in strigolactone mutants, by postulating that the mode of action of strigolactone is to reduce the accumulation of PIN1 on the PM, thus making canalization more difficult to achieve, requiring a higher initial flux of auxin to drive the positive feedback loop. The model also explains the requirement for a competing auxin source for strigolactone-mediated bud inhibition [17],[18].

One attractive feature of this model is that it establishes a regulatory framework underpinning the ability of plants to balance growth across the shoot system, integrating local (e.g., light quality) and systemic (e.g., nutrient limitation) information, through bud–bud competition. However, the validity of this model remains controversial because of the substantial body of evidence consistent with the hypothesis that strigolactones act locally and specifically in buds to inhibit their growth, by up-regulating the transcription of the TCP family transcription factor, *BRC1*, which is known to be required for stable bud inhibition [14],[25],[26].

In this article, we use computational modelling to generate predictions that allow these alternative hypotheses for strigolactone action to be distinguished. Our results strongly support the auxin transport canalization model for shoot branching control. Specifically, we demonstrate that strigolactone treatment can either inhibit or promote shoot branching, depending on the auxin transport status of the treated plants. This is difficult to reconcile with direct bud inhibition by strigolactone. In contrast, these responses can be explained if strigolactones act by regulating PIN1 removal from the PM of cells in the shoot. Consistent with this mode of action, we show that a rapid primary response to strigolactone is clathrin-dependent PIN1 depletion from the PM.

## Results

The auxin transport canalization-based model for shoot branching control places the auxin transport network as a central component of systemic growth co-ordination in plants. To test this idea further, we investigated the relationship between auxin transport, PIN1 accumulation, and shoot branching in Arabidopsis mutants. In roots, many PM proteins, including PIN1, cycle between the PM and endocytic compartments (reviewed in [27]). This process involves GNOM (GN), a Brefeldin A (BFA)–sensitive ARF–GEF that mediates exocytosis [28], and TRANSPORT INHIBITOR RESISTANT3 TIR3/BIG [29],[30], a putative E3 ligase [31] that co-localises in detergent-resistant membrane fractions with PIN1 and the 1-*N*-naphthylphthalamic acid-binding auxin transporter, ABCB19 [32]. Mutation in *GN* or *TIR3* causes increased shoot branching soon after floral transition [20],[33], whereas mutation in *ABCB19* and its homologue *ABCB1* have no discernible effect at this stage [34].

**Figure 1:**
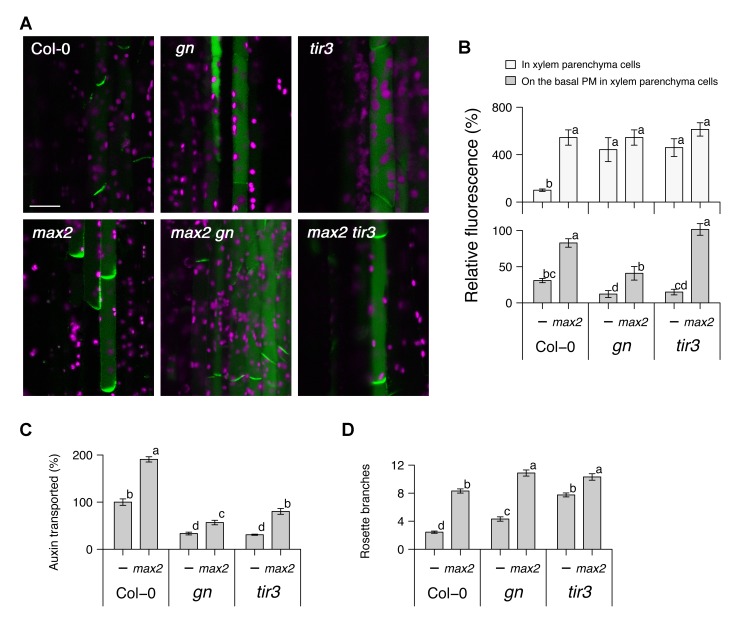
Genetic interactions between *max2*, *gn*, and *tir3*. (A) Micrographs and (B) their quantitative analysis of longitudinal sections from inflorescence stems of 6-wk-old soil-grown Arabidopsis plants harbouring *PIN1:PIN1–GFP* in either the wild-type, *gn*, or *tir3* genetic background, with or without *max2*. (C) Stem polar auxin transport levels and (D) the number of rosette branches in plants of the above genotypes grown on soil. In (A), green shows the PIN1–GFP signal, predominantly localised to xylem parenchyma cells, and magenta shows autofluorescence of chloroplasts; scale bar: 20 µm. In (B), the whole-cell signal (light grey) and signal localised to the basal PM (dark grey) are shown as means ± s.e.m. of nine xylem parenchyma cells in three to four plants as a percentage to the whole-cell signal of the wild-type; samples were compared by Tukey's test. In (C), stem segments were excised from 6-wk-old plants and incubated in liquid medium containing 1 µM [^14^C] IAA, and the amount of radiolabelled auxin transported over a period of 6 h was measured and converted to the percentage of wild-type; means ± s.e.m. of 16 segments are shown; samples were compared by Tukey's test. (D) shows means ± s.e.m. of 16 8-wk-old plants; samples were compared by Steel–Dwass test. Results presented are typical of at least two independent experiments.

To investigate the relationship between increased shoot branching, strigolactone action, and PM PIN1 accumulation, we compared *gn*, *tir3*, and the strigolactone-signalling mutant *max2* in single and double mutant combinations for these phenotypes (Figure 1). PM accumulation of PIN1 was assessed in hand sections through main inflorescence stems of 6-wk-old plants harbouring a *PIN1:PIN1-GFP* transgene. All the mutants tested show increased overall fluorescence in xylem parenchyma cells (Figure 1A,B). For *gn* and *tir3*, reduced PM PIN1 was associated with reduced auxin transport and increased shoot branching (Figure 1A–D). The reduction in auxin transport observed in *gn* and *tir3* is of a similar magnitude to that reported by Okada et al. [4] for the *pin1* mutant and confirmed in our conditions (Figure S1). In contrast, *max2* had increased shoot branching, with increased PM PIN1 and increased auxin transport (Figure 1A–D), consistent with previous reports [19]. Double mutants between these two classes had at least partially additive phenotypes (Figure 1A–D), with higher shoot branching than the single mutants, and intermediate levels of auxin transport and PM PIN1, except in the *max2 tir3* double mutant, where PM PIN1 levels were similar to *max2*. These results suggest that while GN, TIR3, and strigolactones are all involved in PM PIN1 accumulation, their modes of action are at least partially independent.

### Computational Modelling of Strigolactone Action

Many biological behaviours are the outcome of interlinked feedback regulation acting recursively. Consequently, these behaviours are difficult to understand by intuitive interpretation of biological observations. Formalisation of these systems through mathematical modelling and computer simulation can link mechanistic hypotheses for their action to emergent higher order behaviour and thereby increase understanding of the underlying mechanisms. We previously presented a computational model for shoot branching control, based on the auxin transport canalization hypothesis described above [20]. This model can account for the phenotypes of *gn* or *tir3* mutation, and strigolactone treatment, if their actions are to reduce insertion or enhance removal of PIN1 from the PM [20]. The heart of the model is Equation 1, which encapsulates the positive feedback of auxin transport canalization. PIN1 levels in the membrane depend on both insertion, captured by a rate (ρ) proportional to the flux of auxin across the membrane, and removal, captured by a rate (*μ*) (for full details, see [20]):
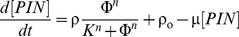
(1)


To dissect further which parameters in this model might be affected by *max*, *gn*, and *tir3* mutation, we set “wild-type” values of the parameters and ran simulations with individual input values for each parameter in turn, changed around the wild-type value. The simulation outputs are summarised for shoot branching levels, polar auxin transport levels, and PIN protein levels in Table 1. Of the 14 parameters, 13 were able to capture branchy phenotypes with some input values. Of these, only three captured both branchy phenotypes and altered levels of polar auxin transport. These were ρ (the PIN insertion constant), μ (the PIN removal constant), and T (the polar transport coefficient—the efficiency with which each PIN protein transports auxin). To match the biological data, GN and TIR3 activity should be explained by a parameter whose reduction can elevate branch numbers, reduce polar auxin transport, and reduce PIN1 accumulation (Figure 1). Only ρ (the PIN insertion constant) satisfies these criteria (Table 1). Similarly, strigolactone/MAX activity should be explained by a parameter whose reduction can increase shoot branching, polar auxin transport, and PIN1 accumulation (Figure 1). Only μ (the PIN removal constant) satisfies these criteria (Table 1).

**Table 1:** Parameter space exploration in a computational model for shoot branching.

				Output					
Parameter^a^		Input		Number of branches^c^		PAT level^d^^,^ e		PIN level^d^^,^ f	
Name	Symbol	WT^b^	Range	Range	Trend^g^	Range	Trend^g^	Range	Trend^g^
PIN removal constant	*μ*	1.8	0.15 to 3.6	1 to 10	<$>\scale 60%\raster="rg1"<$>	51 to 1,095	<$>\scale 60%\raster="rg1"<$>	50 to 1,200	<$>\scale 60%\raster="rg1"<$>
PIN insertion constant	*ρ*	2.7	0.15 to 3.6	0 to 7	<$>\scale 60%\raster="rg1"<$>	7 to 131	↗	4 to 132	↗
Polar transport coefficient	T	0.5	0.3 to 2.6	1 to 10	↗	61 to 496	↗	100 to 100	c
Base PIN production rate	*ρ_0_*	0.1	0.088 to 0.226	2 to 8	↗	100 to 104	c	100 to 105	c
Hill exponent	n	4.5	1.5 to 13	0 to 10	<$>\scale 60%\raster="rg1"<$>	99 to 100	c	100 to 100	c
Hill saturation coefficient	K	0.5	0.08 to 0.77	1 to 10	<$>\scale 60%\raster="rg1"<$>	100 to 100	c	100 to 100	c
Diffusion coefficient	D	0.02	0.014 to 0.06	2 to 9	↗	99 to 104	c	100 to 100	c
Target auxin concentration	H	10	4 to 27	0 to 10	↗	100 to 100	c	100 to 100	c
Residual auxin concentration	Hr	3.5	1.4 to 3.7	2 to 7	<$>\scale 60%\raster="rg1"<$>	100 to 100	c	100 to 100	c
Auxin production rate	*σ*	10	0.5 to 12	2 to 10	<$>\scale 60%\raster="rg1"<$>	100 to 100	c	100 to 100	c
Residual auxin production rate	*σr*	10	0.32 to 20.56	2 to 8	<$>\scale 60%\raster="rg1"<$>	100 to 100	c	100 to 100	c
Threshold bud-activation flux	Φ*th*	2	0 to 2.3	2 to 10	<$>\scale 60%\raster="rg1"<$>	100 to 100	c	100 to 100	c
Auxin turnover rate	*ν*	0.01	0.005 to 0.12	2 to 10	↗	100 to 100	c	s to 100	c
Auxin turnover rate in the root	*νroot*	1	0.5 to 12	2 to 2	c^h^	100 to 100	c	100 to 100	c

a The time step was set to 2,000, the number of lateral buds was set to 10, and other conditions were according to Prusinkiewicz et al. (2009) [20].

b Values for wild-type simulation.

c Wild-type simulation, 2; maximum, 10.

d Percent of wild-type simulation.

e The simulated polar auxin transport level.

f The basally localised PIN protein level in the second most basal metamer (phytomer) of the main stem.

g Downward arrow, negative trend; upward arrow, positive trend; c, constant and no trend (less than 5% change).

h After testing various input values, we concluded that this parameter did not affect the shoot branching level within a valid input range.

**Figure 2:**
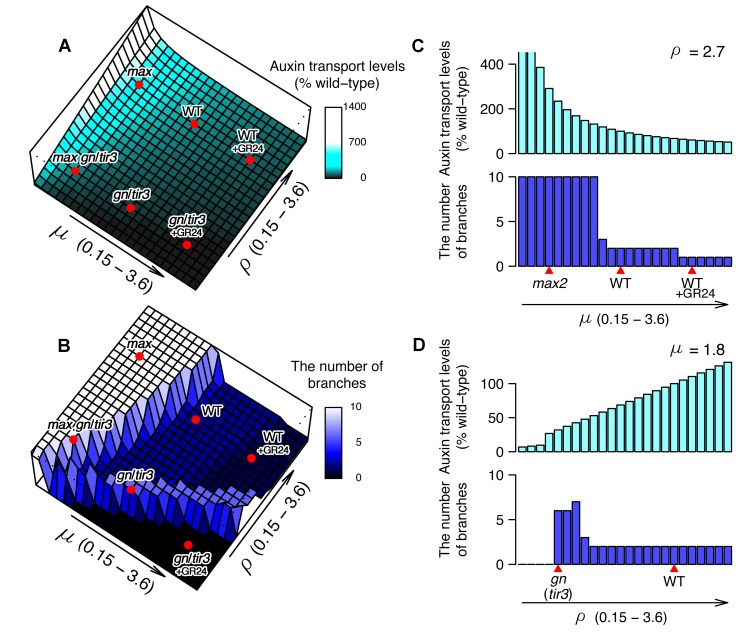
Landscapes of simulated polar auxin transport and shoot branching. (A) Simulated polar auxin transport levels and (B) shoot branching levels shown as heights on the μ–ρ plane, where μ is the removal constant and ρ is the insertion constant for PM PIN1 [20]. Cross-sectional views of (A) and (B) at the simulated wild-type ρ value (C) and at the simulated wild-type μ value (D). Relative positions (red marks) of simulated genotypes in the μ–ρ plane are shown.

To understand better the relationship between the parameters and simulation outputs, we plotted two 3-dimensional graphs that show PAT (Figure 2A) and shoot branching (Figure 2B) levels as heights on the μ–ρ plane. The relationship between polar auxin transport levels and μ–ρ was relatively simple: as PIN removal (μ) decreased and PIN insertion (ρ) increased, the polar auxin transport level gradually increased, resulting in a smooth slope (Figure 2A,C,D). In contrast, the relationship between shoot branching level and μ–ρ was more complex: as PIN removal (μ) decreased, the shoot branching level increased, creating a plateau of high branching at low μ values. However, as PIN insertion (ρ) decreased the branching level increased, even when PIN removal (μ) was quite high, resulting in a ridge of high branching (Figure 2B). High branching on the low μ (low PIN removal) plateau is caused by easy establishment of canalization of auxin transport from bud to stem, with low initial auxin fluxes able to establish canalization through positive feedback, making buds difficult to inhibit. High branching along the low ρ (low PIN insertion) ridge is caused by low auxin efflux from active shoot apices, such that a larger number of active apices are needed to supply sufficient auxin to the main stem to prevent activation of further buds. The profiles for branch number at any one μ or ρ value made much more abrupt transitions than for auxin transport levels (Figure 2C,D), with mostly high or low branch numbers, and only narrow regions of parameter space giving intermediate branch numbers. This is because branch activation in the model is triggered by canalization of auxin transport out of the simulated bud and the positive feedback inherent in the canalization process produces switch-like behaviour [20].

To capture the behaviour of strigolactone biosynthesis mutants such as *max4* or strigolactone-signalling mutants such as *max2*, we assigned a low value to μ, conditioning slow PIN removal. This resulted in higher levels of both polar auxin transport and branching compared with those of the defined wild-type (Figure 2A,B), consistent with biological results (Figure 1 and [17],[19]). Similarly we simulated the *gn* or *tir3* mutations as a low ρ value, conditioning low PIN insertion, resulting in a lower level of polar auxin transport and a higher level of branching (Figure 2A,B), as observed in biological experiments (Figure 1 and [20],[33],[35]). To simulate addition of the synthetic strigolactone, GR24, we increased the value of μ (increasing PIN removal), which gave slightly lower polar auxin transport and shoot branching levels compared to the defined wild-type (Figure 2A,B), consistent with published biological data [17]. When the low μ value of *max* and the low ρ value of *gn* or *tir3* were simultaneously applied, the model predicts moderate polar auxin transport levels and high branching, consistent with biological results (Figure 1 and [20]). Thus, single parameter changes in the model capture the phenotypes of wild-type, single and double mutants, and where known, their responses to GR24. Furthermore, the relative magnitude of the responses to GR24 in different genetic backgrounds and with respect to branching versus auxin transport is also captured.

### Validating Model Predictions

**Figure 3:**
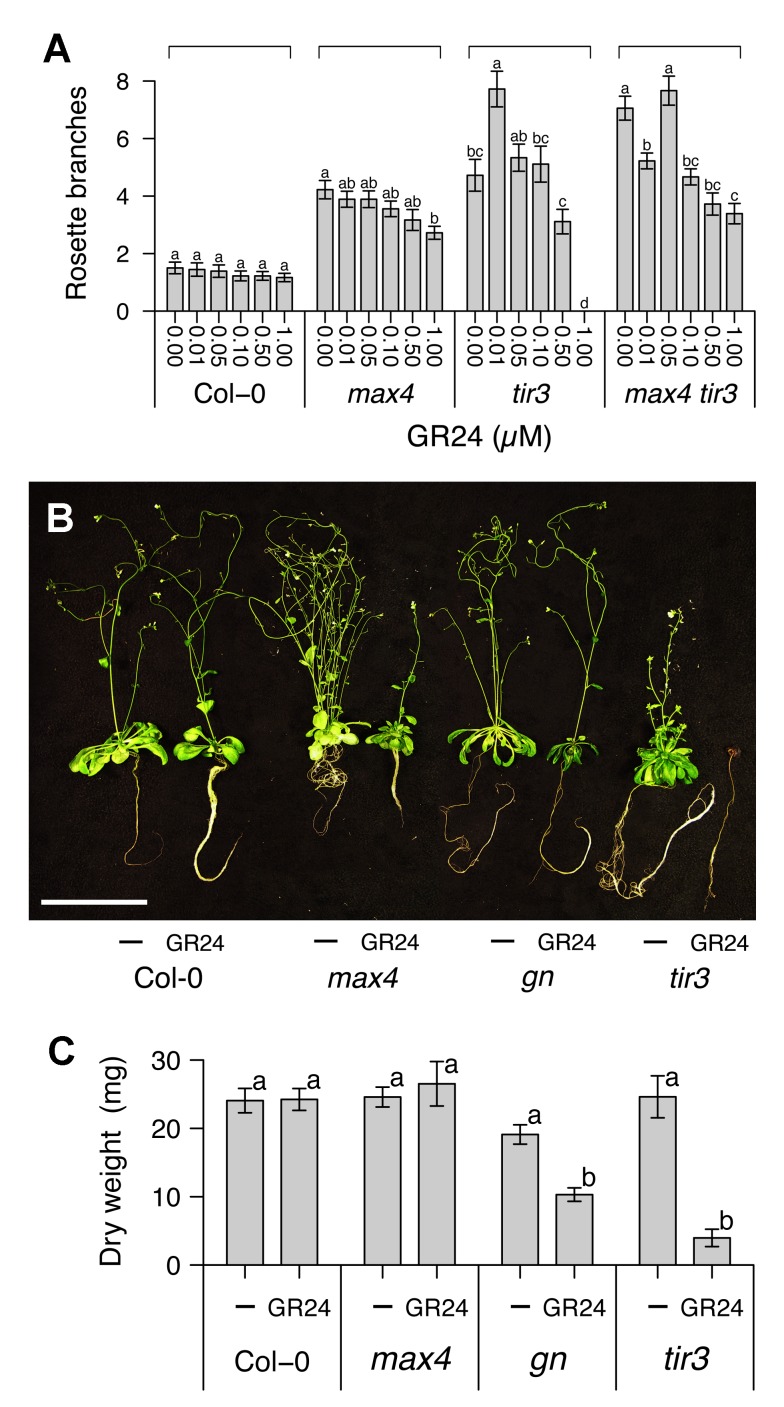
Combinatorial effect of GR24 and *tir3* on shoot branching and growth. (A) The number of rosette branches of wild-type, *max4*, *tir3*, and *max4 tir3* Arabidopsis plants grown for 8 wk in glass jars on agar medium supplemented with the indicated concentrations of GR24. (B) Images and (C) dry weights of wild-type, *max4*, *gn*, and *tir3* Arabidopsis plants grown for 8 wk in glass jars on agar medium supplemented with the vehicle control or 5 µM GR24. In (A), means ± s.e.m. of 18 plants are shown; samples in each genotype were compared by Steel–Dwass test. In (B), scale bar: 5 cm. In (C), means ± s.e.m. of 15 plants are shown; samples were compared by Tukey's test.

This analysis led to an interesting and counterintuitive prediction. The dose-response curve of *max4 tir3* branch number to GR24 is predicted to have two peaks, which lie on the low PIN removal (μ) plateau and low PIN insertion (ρ) ridge (Figure 2B). To test this prediction, we grew wild-type, *max4*, *tir3*, and *max4 tir3* plants for 8 wk on agar-solidified medium supplemented with GR24 ranging from 10 nM to 1 µM (Figure 3A). As previously shown [17], in both wild-type and *max4*, GR24 reduced branching levels monotonically, although this effect was not statistically significant in the wild-type. In contrast, in *tir3*, GR24 significantly elevated branching levels at 10 nM, and reduced branching at higher concentrations, with 1 µM resulting in very poor growth. In *max4 tir3*, 10 nM GR24 reduced branching levels, but 50 nM GR24 restored branching levels to those of untreated plants and higher concentrations reduced them again. This latter part of the curve was shifted compared to the *tir3* alone, with branched plants produced at 1 µM, a concentration that severely inhibits growth in *tir3* mutants. Therefore, GR24 did not simply inhibit but also promoted shoot branching depending on the concentration and the genetic background of the treated plant. These results validate the predictions of the model with the minor modification that the effects of *tir3* mutation on PIN insertion (ρ) suggest that it is placed on the low μ slope of the low ρ ridge, rather than at its summit, as proposed in Figure 2.

As well as the unusual dose–response relationships, the parameter space exploration predicts no branching at high PIN removal (μ) and low PIN insertion (ρ), caused by insufficient auxin transport to support bud growth. In the dose–response experiments described above, 1 µM GR24 severely affected the growth of the *tir3* mutant. To explore the response of *tir3* and *gn* mutants to high levels of GR24 in more detail, we grew wild-type, *max4*, *gn*, and *tir3* plants for 8 wk on agar-solidified medium containing 5 µM GR24, or an equivalent volume of solvent. GR24 affected the overall vigor of *gn* and *tir3* plants, such that their total dry weights were significantly reduced compared to untreated controls (Figure 3B,C). This effect was particularly noticeable in *tir3* plants (Figure 3B,C), which often did not survive to maturity in the presence of 5 µM GR24. GR24 had no effect on dry weight in wild type or *max4* (Figure 3C). Thus *gn* and *tir3* shoots are hypersensitive to GR24.

### Strigolactone Reduces PM PIN1 by a Clathrin-Dependent Mechanism

**Figure 4:**
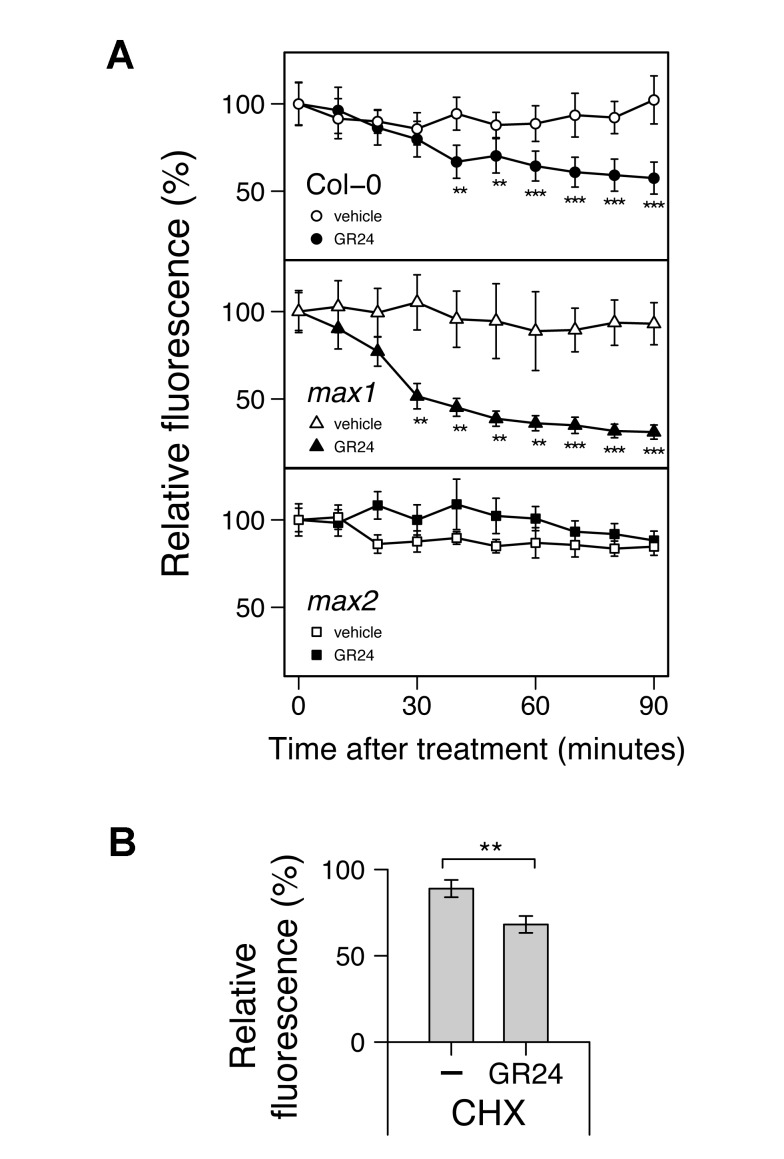
Effect of GR24 on PIN1 protein dynamics in inflorescence stems. (A) Real-time monitoring of GR24-induced PIN1 depletion from the basal PM in wild-type, *max1*, or *max2*. (B) Effect of the protein synthesis inhibitor cycloheximide (CHX) on GR24-induced PIN1 instability in wild-type. The GFP signal in longitudinal sections from inflorescence stems of 6-wk-old soil-grown plants harbouring *PIN1:PIN1–GFP* in either the wild-type, *max1*, or *max2* genetic background was monitored. Sections were mounted with the vehicle control or 5 µM GR24. For (B), sections were pretreated with 50 µM CHX for 30 min before addition of the vehicle control or 5 µM GR24 for 60 min. Means ± s.e.m. of the basal PM region of 7–9 cells are shown as a percentage of the value just after mounting. The vehicle control versus GR24-treated samples were compared by one-tailed Student's test. Results presented are typical of three independent experiments.

These data strongly support the hypothesis that strigolactones increase the removal of PIN1 from the PM, and indeed we have previously shown that GR24 treatment reduces PIN1 abundance in xylem parenchyma cells within 6 h in a MAX2-dependent manner [17]. To investigate the dynamics of this process in more detail, we prepared hand sections of stems of different genotypes harbouring the PIN1–GFP fusion, as described above, and recorded basal PM PIN1 levels every 10 min over a 90-min period. PIN1 was significantly reduced by the addition of 5 µM GR24 within 40 min in wild-type plants and within 30 min for *max1* plants (Figure 4A). As expected, no significant difference was observed in *max2* mutants (Figure 4A). We also examined wild-type sections treated with 50 µM cycloheximide for 30 min before a 60-min incubation with 5 µM GR24 or the vehicle control. GR24-induced depletion of PM PIN1 level was unaffected by cycloheximide treatment (Figure 4B), suggesting that this process is independent of new protein synthesis.

**Figure 5:**
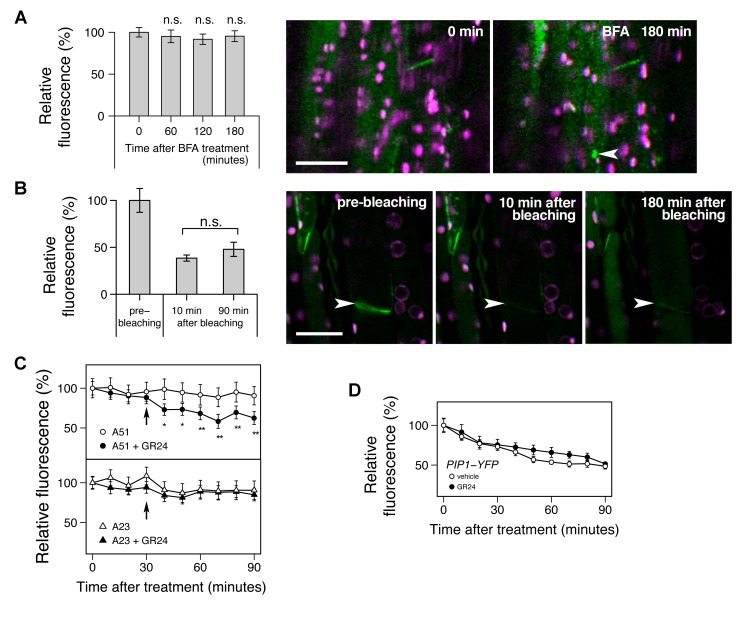
Characterisation of strigolactone-induced PIN1 protein instability in inflorescence stems. (A) Effect of the vesicle trafficking inhibitor brefeldin A (BFA) on PIN1 protein. (B) Lack of recovery of PIN1 signal after photobleaching. (C) Effect of the clathrin-dependent endocytosis inhibitor A23 on GR24-induced instability of PIN1 protein. (D) Effect of GR24 on stability of a PM-localised protein other than PIN1. In (A–C), the GFP signal in longitudinal sections from inflorescence stems of 6-wk-old soil-grown plants harbouring *PIN1:PIN1–GFP* was monitored up to 180 min after mounting with 50 µM BFA for (A), before and up to 180 min after photobleaching for (B), or up to 90 min after mounting prior to 30-min pretreatment of 50 µM A23 or its inactive analogue A51 before addition of the vehicle control or 5 µM GR24 (indicated by the arrow) for (C); means ± s.e.m. of the basal PM region of 5–9 cells are shown as a percentage to the value just after mounting; comparison was performed between samples just after mounting and either 60, 120, or 180 min after mounting by one-tailed paired *t* test for (A), between samples 10 min and 90 min after photobleaching by one-tailed paired *t* test for (B), or between the vehicle control and GR24-treated samples by one-tailed Student's test at each time point for (C). In micrographs of (A) and (B), green shows the PIN1–GFP signal, and magenta shows autofluorescence of chloroplasts; scale bar: 20 µm; an arrowhead indicates PIN1-rich compartment in (A), or a bleached region of the basal PM in (B). In (D) the YFP signal in longitudinal sections from inflorescence stems of 6-wk-old soil-grown plants harbouring *UBQ10:PIP1–YFP*, which encodes a fluorescence-tagged aquaporin protein, was monitored up to 90 min after mounting with the vehicle control or 5 µM GR24; means ± s.e.m. of the basal PM region of seven cells are shown as a percentage of the value just after mounting; the vehicle control versus GR24-treated samples were compared by Student's test at each time point. Results presented are typical of at least two independent experiments.

Depleted PM PIN1 could in principle result from either increased removal or reduced insertion of PIN1. In roots, there is good evidence that many membrane proteins, including PIN1, cycle rapidly between the PM and endomembrane compartments [28]. The removal of these proteins from the PM is mediated by clathrin-dependent endocytosis, which is often assessed by quantifying the accumulation of BFA-induced endomembrane compartments [36],[37]. BFA inhibits the activity of ARF–GEFs such as GN, preventing recycling of proteins back to the PM, resulting in their depletion from the PM and accumulation in endomembrane compartments. We treated stem segments with 50 µM BFA for 3 h, but we observed that this treatment had no significant effect on the amount of PIN1 on the basal PM (Figure 5A), and relatively few PIN1 containing compartments were identified. Only 9 of 29 cells examined with optical sectioning throughout the *z*-axis contained a compartment. This contrasts to results previously described for PIN1 in roots, where after 90 min treatment with 25 µM BFA, the mean number of BFA compartments per cell was more than 1 [37]. These results suggest that either PIN1 endocytosis in stems is BFA-sensitive (for tissue-dependent BFA effects, see [38]) and/or PIN1 cycles only slowly in stem segments. To assess the rate of PIN1 allocation to the PM, we used fluorescence recovery after photobleaching. In root cells, after photobleaching total PIN1 signal from a cell, nonpolar PM PIN1 was detected after 100 min [39]. We bleached only the basal PM PIN1 of xylem parenchyma cells, and no significant fluorescence recovery was detected 90 min after bleaching, with little visible effect even after 3 h (Figure 5B), suggesting low insertion rates for PIN1 from either intracellular stores or de novo synthesis. This suggests that at steady state, either cycling rates in stems are low or the fraction of PIN1 in intracellular compartments is very low.

To test whether GR24-triggered PIN1 depletion is clathrin-dependent, we determined the effect of the clathrin inhibitor, A23 [40]. A23 treatment alone had no effect on PIN1 levels, providing further evidence for a low rate of insertion of PIN1 or a low intracellular fraction. However, in the presence of A23, including a 30-min pretreatment, the ability of GR24 to deplete PM PIN1 was abolished (Figure 5C), whereas when treated with the structurally related but inactive control A51, GR24 triggered PIN1 depletion from the PM as previously observed (Figure 5C). These results suggest that a rapid, nontranscriptional mode of action of strigolactone is to promote a clathrin-mediated step in PIN1 depletion. Indeed in this experiment, statistically significant depletion of basally localised PIN1 was observed within 10 min.

As described above, in roots there is rapid constitutive cycling of PIN1 between the PM and the endomembrane system. However, this cycling is not specific but rather reflects general cycling of many proteins. Treatments that affect PIN1 levels at the PM, such as auxin, BFA, and A23 treatment, also affect many other membrane proteins, such as water channel proteins of the PIP1 and PIP2 families [28],[36],[37]. Thus in the root, a major contributor to PIN1 behaviour is general trafficking activity. We therefore tested the specificity of the effects of GR24 on PIN1 PM levels in shoots by assessing its effects on PIP1 [41]. PIP1 levels on the PM were less stable over time than PIN1 levels and were halved after 90 min, regardless of the presence or absence of GR24 (Figure 5D), indicating that GR24 has no effect on PIP1 levels. These results suggest that the effect of strigolactone on PIN1 PM levels in stems is more specific than known mechanisms regulating PM PIN1 levels in roots.

### Strigolactone Action in Roots

The apparent specificity of strigolactone effects on PM PIN1 in shoots raises interesting questions concerning the effect of strigolactones on roots. Various aspects of root development, such as primary root length, lateral root development, and root hair elongation, have recently been shown to be modulated by strigolactones [42]–[44]. The role of auxin transport in these phenotypes is unclear, but there is some evidence to suggest that they are at least in part mediated by differences in auxin transport, either locally in the root or systemically from the shoot.

**Figure 6:**
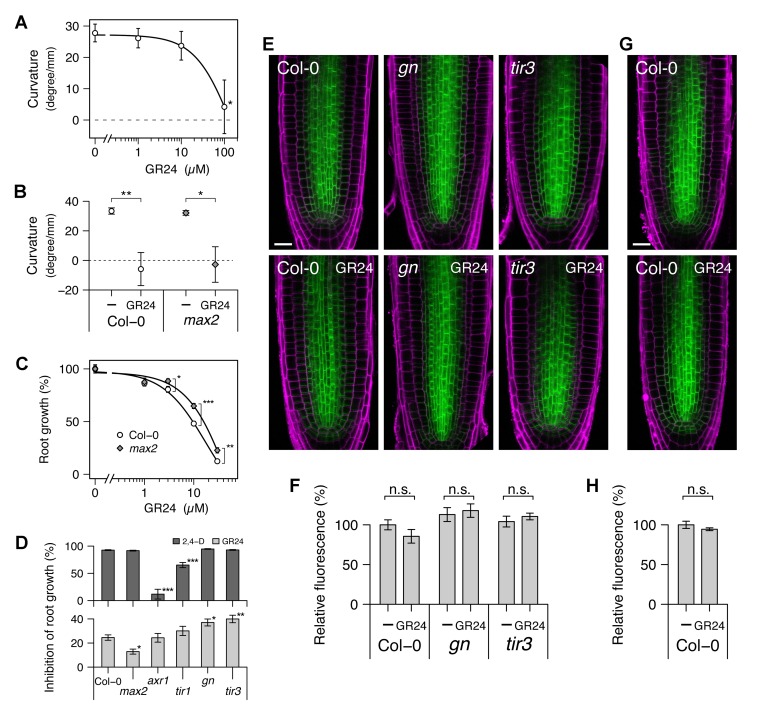
Effects of GR24 on development and PIN1 localisation in roots. (A) Dose–response of gravitropic root growth to GR24 in wild-type seedlings. (B) Gravitropic root growth in wild-type and *max2* seedlings treated with the vehicle control or 100 µM GR24. (C) Dose–response of root growth to GR24 in wild-type and *max2* seedlings. (D) Root growth inhibition by 0.1 µM 2,4-D and 5 µM GR24 in seedlings of various genotypes. (E, G) Micrographs and (F, H) their quantitative analysis of longitudinal optical sections from the primary root. In (A–D), all seedlings were grown for 3 d on hormone-free medium and were preincubated for 24 h on medium containing the vehicle control or relevant hormone before the observation of root growth; means ± s.e.m. of 8–12 seedlings are shown. In (A), the vehicle control versus GR24-treated samples were compared by Shirley–Williams test. In (B), the vehicle control versus GR24-treated samples were compared by Wilcoxon's test in each genotype. In (C), wild-type versus *max2* samples were compared by Student's test at each concentration of GR24; the significant effect of GR24 at 1 µM or higher in both wild-type and *max2* was detected by Williams test at *p*<0.01 (not shown in the graph). In (D), the percentage of each vehicle control-treated sample is shown; wild-type versus mutant samples were compared by Dunnett's test. In (E–H), 4-d-old Arabidopsis seedlings harbouring *PIN1:PIN1–GFP* in either the wild-type, *gn*, or *tir3* genetic backgrounds were treated with the vehicle control or 10 µM GR24 for 12 h (E, F) or 48 h (G, H). In (E) and (G), green colour shows the PIN1–GFP signal, and magenta colour shows cell wall counterstained with propidium iodide; scale bar: 20 µm. In (F) and (H), average intensity of the PIN1–GFP signal in the stele region was measured for each seedling; means ± s.e.m. of three seedlings are shown; in each genotype, the vehicle control versus GR24-treated samples were compared by Student's test. In (F), for each treatment, there was no significant difference (*p*>0.05) between wild-type and either *gn* or *tir3* samples compared by Dunnett's test (not shown in the graph).

To investigate the relationship between auxin transport and the effects of GR24 on roots, we grew Arabidopsis seedlings for 3 d on agar medium without exogenous hormones, preincubated them for 24 h with various concentrations of GR24, and then observed their elongation over the next 24 h. In the wild-type, two responses were found: agravitropic root growth and root growth inhibition (Figure 6; also see [42]). With respect to effective doses, agravitropic root growth required very high concentrations of GR24 (greater than 10 µM, Figure 6A), and there was no significant difference between wild-type and *max2* mutants in response to 100 µM GR24 (Figure 6B). The very high levels of GR24 needed for this effect and lack of dependence on MAX2 led us to conclude that it is of limited physiological relevance. In contrast, as previously shown [42], root elongation was inhibited by more physiologically relevant levels of GR24, with GR24 levels between 3 and 30 µM having a significantly weaker effect on *max2* than on wild-type, although dose-dependent inhibition was observed in both these genotypes (Figure 6C). Thus, root growth inhibition by GR24 is partially MAX2-dependent.

To assess the involvement of auxin in GR24-induced root growth inhibition, we measured root growth in seedlings treated with 0.1 µM 2,4-D, a synthetic auxin, or 5 µM GR24, comparing wild-type, *max2*, *gn*, *tir3*, and the auxin signalling mutants *axr1* and *tir1* (Figure 6D) [45],[46]. The *max2* mutant responded to 2,4-D as wild-type and showed resistance to GR24; *axr1* and *tir1* showed resistance to 2,4-D and responded normally to GR24; *gn* and *tir3* responded normally to 2,4-D but showed mild hypersensitivity to GR24. Thus, as in the shoot, there is an interaction between GR24 and GN/TIR3.

To test the effects of GR24-treatment and *gn* and *tir3* mutation on PIN1 protein levels in roots, we observed the root tips of 4-d-old Arabidopsis seedlings harbouring a *PIN1:PIN1–GFP* transgene in either the wild-type, *gn*, or *tir3* genetic backgrounds after a 12-h incubation with or without 10 µM GR24. Neither GR24-treatment, *gn*, nor *tir3* altered total signal levels or obvious subcellular localisation of PIN1 protein in the root tip (Figure 6E,F). Even after a 48-h incubation, 10 µM GR24 did not alter total signal levels or obvious subcellular localisation in wild-type (Figure 6G,H). These results are consistent with different PIN1 trafficking dynamics in roots compared to shoots, such that relatively modest increases in strigolactone-triggered PIN1 PM depletion have a much more dramatic effect in the shoot compared to the root.

## Discussion

### Auxin, Strigolactone, and the Self-Organisation of the Shoot System

In the 1930s Thimann and Skoog established that auxin synthesized in active shoot apices is transported down the main stem and inhibits the activity of axillary shoot apices in subtending leaf axils [6],[7]. However, it was rapidly discovered that auxin acts indirectly to inhibit axillary bud growth, and furthermore there was a fundamental paradox in auxin behaviour. On the one hand, auxin inhibited the activity of axillary buds, but on the other, its synthesis and export from active apices protected them from inhibition by other auxin sources [47]. These classical observations are explicable by the auxin transport canalization based model for shoot branching control. According to this idea, all the meristems in a shoot compete for access to a common auxin transport path down the main stem to the root. Rootward auxin transport from each shoot apex is established by the positive feedback process of auxin transport canalization, the dynamics of which are critically dependent on the strength of the bud as an auxin source, the strength of the stem as an auxin sink, and the dynamics of the positive feedback loop at the centre of the canalization process that connects them. Thus, the auxin transport system in the shoot forms a self-organising network through which all shoot apices communicate by contributing auxin into the system, thereby influencing the ability of other apices to export auxin.

**Figure 7:**
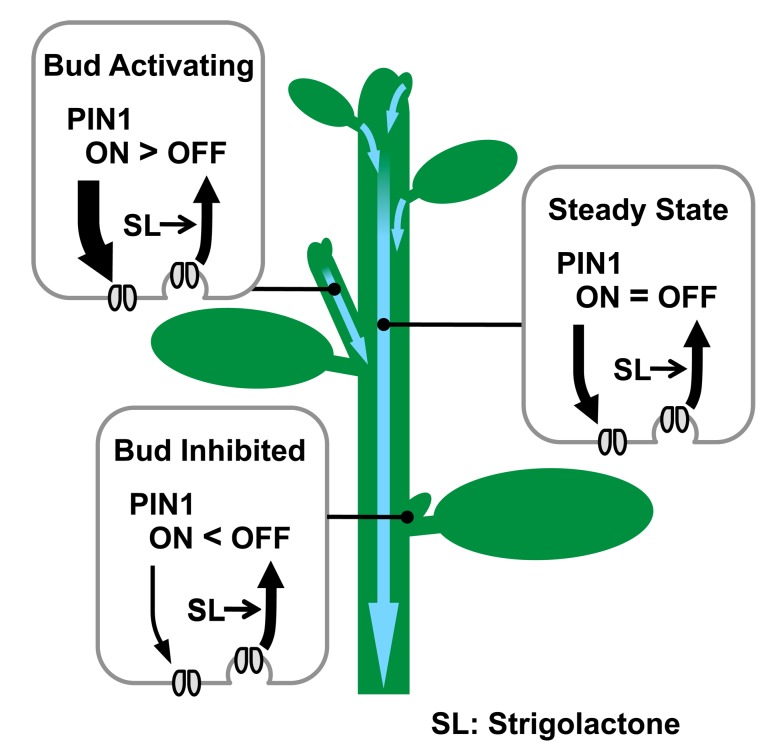
Schematic representation of PIN1 protein regulation by strigolactone and its effect on bud activity. Strigolactone, signalling via MAX2, depletes PIN1 from the PM of cells in the shoot, for example by promoting clathrin-mediated endocytosis. Strigolactone acts systemically, influencing PM PIN1 levels throughout the shoot. In the main stem, PIN1 on the PM is at steady state. In an activating bud, canalization is underway, with rapid PIN1 insertion, outstripping PIN1 removal. In an inhibited bud, PIN1 insertion is slower than PIN1 depletion, such that PIN1 does not accumulate on the PM [20],[23]. Systemically higher strigolactone levels will reduce the number of active buds and the steady-state levels of PM PIN1. Systemically lower strigolactone will have the opposite effect.

This mechanism for shoot branching control is attractive because it explains the classical observations mentioned above and readily supports the integration of both local and systemic factors in balancing growth distribution across the shoot. However, the idea remains controversial, largely due to different ideas about the mechanism of action of another branch-regulating hormone, strigolactone. One hypothesis, generally referred to as the second messenger hypothesis, posits that auxin in the main stem up-regulates the production strigolactone, which moves into the axillary buds and inhibits their growth by locally up-regulating transcription of the TCP family transcription factor *BRC1*, which is known to be required for stable bud inactivation [14],[25],[26]. A second hypothesis assumes that axillary bud activity is regulated by the auxin transport canalization-based mechanism described above and that strigolactone acts by modulating auxin transporter accumulation, thereby modulating the ease with which axillary buds can establish active auxin transport into the main stem (Figure 7) [17],[19],[20],[23]. Thus the mechanism of strigolactone action and the mechanism of auxin-mediated bud inhibition are tightly intertwined, representing two different scenarios for the systemic coordination of growth across the shoot system.

The results presented here strongly support the second hypothesis. A particularly striking illustration of this is the ability of strigolactone to promote shoot branching in the *tir3* mutant background (Figure 3), which is difficult to explain if strigolactones act as direct inhibitors of bud growth but is a prediction of the model in which strigolactones act to modulate auxin transport (Figure 2). It should be noted that the two models can easily be reconciled. For example, the primary mode of action for strigolactone could be on PIN1 accumulation, and the resulting effects on auxin transport could in turn influence *BRC1* transcript levels. Up-regulation of *BRC1* by strigolactone addition to pea buds has been shown to be independent of new translation, but so far it has only been measured after 6 h [26],[48], and no such up-regulation was detected in a similar experiment in rice after 3 h of treatment [49]. In contrast, in Arabidopsis stems, an effect on PIN1 accumulation was observed within 10–40 min of strigolactone application (Figures 4 and 5), and this effect is also independent of new protein synthesis. It is therefore possible that *BRC1* transcript changes are downstream of changes in PIN1 accumulation, and the role of BRC1 could be to stabilise bud inactivation caused by low auxin export. Some stabilizing system to maintain bud inactivity seems intuitively important, because bud activation by the positive feedback inherent in canalization is highly likely to be triggered by stochastic variation in the system.

These two models differ in that in the canalization model, strigolactones act systemically on the auxin transport network, including in the bud (Figure 7), whereas in the second messenger model, they act locally and specifically in buds. The systemic expression of MAX2 in xylem-associated cells and the effect of strigolactone on PIN1 accumulation in the main stem are consistent with systemic action. This mode of action allows strigolactones to modulate bud–bud competition systemically, for example in response to nutrient deprivation [13]. In this context, systemic strigolactone levels determine how many buds can activate, but they do not determine which buds activate. This can be regulated by local factors such as light levels [50]. Thus, both local and systemic modifications to the auxin transport network can integrate different environmental inputs to direct resource allocation across the plant body. A more direct mode of action for strigolactone locally in buds does not have this interesting property. However, the two models, and indeed others, are mutually compatible and could operate in parallel with either species-specific and/or environment-specific variation in their relative importance.

### Molecular Mechanism of Strigolactone Action

Little is known about the molecular mechanism of strigolactone action. Only two genes have been implicated in strigolactone signalling. These are *MAX2*, which encodes an F-box protein presumed to be required for the strigolactone-regulated ubiquitination of one or more specific target proteins, and D14, which encodes an α/β hydrolase protein that binds GR24, confers signal specificity to the pathway [51]–[53], and could either act as a receptor or could process strigolactones to form a final bioactive product. The immediate downstream effectors of the pathway are unknown, but the largely nuclear localization of MAX2 [54] and the rapid changes in transcription induced by many F-box-protein–mediated plant hormone signalling pathways [55] have led to an assumption that the primary targets for the strigolactone pathway are also transcriptional. The evidence to support this mode of action is currently quite weak. Few reliable transcriptional readouts for strigolactone response have been identified. These tend to have slow induction kinetics, in the order of several hours, and relatively small fold inductions [26], suggesting that they may be secondary responses or limited to a small proportion of cells. Microarray analysis of Arabidopsis seedlings treated with or without 1 µM GR24 for 90 min shows that 76% of all the GR24-repressible genes are categorised as auxin-inducible [56], and thus these transcriptional effects may be indirectly mediated via changes in auxin distribution.

Consistent with this idea, we have shown that a rapid translation-independent response to stigolactone addition is changes in PM PIN1 accumulation (Figure 4). Thus, at least one immediate early target downstream of MAX2 in the stem is not transcriptional but involves PIN1 depletion from the PM by an A23-sensitive mechanism, such as clathrin-mediated endocytosis [40]. The mechanism by which the substantially nuclear MAX2 influences PM PIN1 is not known. However, our data suggest that it is both quantitatively and qualitatively different from the major PIN1-regulatory systems operating in the root. Several lines of evidence support this conclusion. First, in stems, strigolactone response is independent of TIR3 activity, which has been reported to be required for auxin-induced inhibition of clathrin-mediated endocytosis in roots [30]. Second, the effect of strigolactone on PIN1 depletion from the PM in stems appears to be more specific than the systems operating in roots, since it does not affect the PM levels of PIP1, although we have not excluded targets beyond PIN1. Third, the MAX2-dependent effects of strigolactones on root phenotype are generally less dramatic than those observed in shoots, both with respect to cell biological and whole organ-level phenotypes.

### Strigolactones and Roots

Although more modest than the effects on shoots, long-term effects of GR24 treatment on PIN1 accumulation in the root tip have been detected following 6 d of growth in the presence of 5 µM GR24 [44]. These effects have been correlated with reduced shoot-to-root auxin transport, suggesting that they represent a transcriptional response to low auxin rather than the protein trafficking mechanism we propose here. Consistent with this idea, the accumulation of multiple PIN proteins is affected in these root tips, including in cell layers where MAX2 is not expressed at detectable levels [44],[54]. However, although we found only weak MAX2-dependent root growth inhibition by GR24, this occurred with equal effect in the auxin signalling mutants, *axr1* and *tir1*, suggesting that GR24 reduces root growth at least to some extent independently of auxin concentration-mediated effects. Similarly, in the trafficking mutants, *gn* and *tir3*, GR24 reduced root growth more severely than in wild-type, suggesting some overlap in the mechanism underlying the control of shoot branching by strigolactone and its effects on root growth.

### Conclusions

Computer simulations of shoot growth using our canalization-based model consistently reproduce biological results when strigolactone action is ascribed to a linear process of PIN removal from the PM, independent of PIN insertion and auxin flux. Consistent with this idea, bioimaging of PIN1 protein in inflorescence stems revealed a substantial increase in PIN1 protein in the basal PM in strigolactone mutants. Furthermore, GR24 promoted rapid, translation-independent, MAX2-dependent depletion of PIN1 from the PM through a mechanism sensitive to A23, an inhibitor of clathrin-mediated membrane trafficking. These results are consistent with the hypothesis that strigolactone functions to promote endocytosis of PIN1 from the PM.

It is interesting that the phenotypes affected by the *max* mutations and by strigolactone treatment are generally those where auxin transport canalization has been implicated. In the root tip, canalization is not usually considered to play an important role in PIN accumulation, although auxin-induced changes in the lateralisation of PIN1 in the root endodermis have been described and compared to canalization processes [22]. If the effects of strigolactones on auxin transport are specifically to modulate canalization, then they provide an opportunity to understand better this enigmatic and poorly understood process, which nonetheless provides powerful explanations for complex patterning events in plants and for their impressive developmental plasticity.

## Materials and Methods

### Plant Lines and Growth Conditions

All lines are in the Col-0 background. Experiments involving *max2*, used *max2-3* [57], *max4*, *max4-1* [15], *axr1*, *axr1-3* [58], *tir1*, *tir1-1* [46], *gn*, *gnom^B/E^* [59], and *tir3*, *tir3-101* [60]. Because we found that the *tir3-101* line from a public stock had an additional glabrous mutation besides a C-to-T nonsense mutation at the 3,095th codon of *TIR3*, a *tir3-101* line free from the additional mutation was made and used. For bioimaging, each line homozygous for the *PIN1:PIN1–GFP* transgene cassette [61] was used. For the PIP1 experiments, the *UBQ10:PIP1–YFP* (Wave138Y) fusion line was used [41]. On-soil and axenic growth conditions were as described previously [17].

### Physiological Analysis

For quantifying root growth inhibition and agravitropic root growth, axenic seedlings grown vertically for 3 d on hormone-free agar medium were preincubated for 24 h on vertically placed agar medium containing either only the vehicle, GR24, or 2,4-D. For evaluating the root growth inhibition, the root tip position was recorded just after the preincubation and 24 h after; thus, the length of the primary root grown for the 24 h was obtained. For evaluating the agravitropic root growth, preincubated seedlings were placed horizontally; the root tip position was recorded just after the gravistimulation and every hour up to the next 24 h; thus, the index Curvature, which we defined as the change in root tip angle per the length of grown root within a range between 1 and 3 mm, was calculated. Other physiological experiments were as described previously [17].

### Computer Simulation

All simulations were according to the model of Prusinkiewicz et al. (2009) [20]. For simulating the auxin transport assay (Figure S2), the two most basal metamers in the main stem of the whole plant simulated for 2,000 time steps were used; of these two metamers, the top one provided an initial value of the PIN concentration at the basal face, and the bottom one provided an initial value of the PIN concentration at the apical face. Auxin concentration in the top metamer was assumed to be 10 and constant over time; auxin concentration in the bottom metamer was assumed to be zero initially and change over time according to the Equations 1 and 2 of Prusinkiewicz et al. (2009) [20]. The auxin concentration of the bottom metamer at time step 10 was calculated from those two initial values of the PIN concentrations and converted to the percentage of wild-type. This percentage is shown as the simulated polar auxin transport level.

### Bioimaging

For imaging PIN1–GFP in inflorescence stems, the most basal part of the primary inflorescence stem of 6-wk-old soil-grown plants was longitudinally halved by hand with a razor blade. The cut surface was immediately observed using light microscopy and a Zeiss LSM 710 confocal microscope to identify xylem parenchyma cells according to both the relative position to xylem vessels and the morphology of the cell. With excitation at 488 nm, images containing emission spectra from 490 to 655 nm were then acquired within a single dynamic range. Reference spectra of GFP and chloroplast autofluorescence were obtained using a *PIN1:PIN1–GFP* line in the wild-type background and were used for linear unmixing of the images. For its quantitative analysis, only xylem parenchyma cells that appeared intact and were exposed to the cut surface were taken into account, and the intensity of their unmixed GFP signal was measured in a region of the basal PM that was manually traced. Data were obtained in the same way for real-time monitoring experiments, except that sections were observed with Zeiss LSM 780 confocal microscope. With excitation at 488 nm, images containing emission spectra from 507–550 nm and 593–719 nm were acquired simultaneously in separate channels. Data were obtained in the same way for the PIP1 experiment, except excitation was at 514 nm, and emission spectra were acquired from 518–621 nm and 647–721 nm. For photobleaching experiments, a region of interest (basal PM of xylem parenchyma cell) was selected and bleached using the 488 nm laser at 50% power for 75 iterations. In all experiments, cells from three or more plants were included for each genotype/treatment, and the results presented are typical of at least two independent experiments.

For imaging PIN1–GFP in the root tip, 3- to 5-d-old seedlings incubated for 12 or 48 h on agar medium containing the vehicle or 10 µM GR24 were immersed in 10 µg/ml propidium iodide for 10 min. The primary root was then observed with Zeiss LSM 510 Meta confocal microscope. The GFP signal excited with a 488 nm laser and the propidium iodide signal excited with a 543 nm laser were collected with a 505–550 nm bandpass filter. For its quantitative analysis, the average intensity of the GFP signal was measured in the stele region of each root.

### Statistical Analysis

Based on the assumption that the root angle after gravistimulation and the number of branches do not always follow the normal distribution, nonparametric methods of Wilcoxon, Steel–Dwass, and Shirley–Williams were used. Otherwise parametric methods of Student, Tukey, Dunnett, and Williams were used. Unless otherwise stated, statistical results of two-tailed tests are shown in graphs in the conventional manner. In Steel–Dwass' and Tukey's tests, different letters denote significant differences at *p*<0.05. In other tests, no marks or n.s. indicate not significant, and significant differences are indicated by asterisks as follows: *p*>0.05; * *p*<0.05; ** *p*<0.01; *** *p*<0.001.

## Supporting Information

Figure S1 Auxin transport through inflorescence stem segments of *pin1* mutants is strongly reduced. Auxin transport in *pin1* mutant stem segments was assessed as previously described [19]. The mean amount of apically supplied radiolabelled auxin (counts per minute) transported to the basal end of stem segments is shown, ± the standard error or the mean, *n* = 20. These results are consistent with previous reports [4].
(DOC)

Figure S2 An overview of experimental and simulation designs of the auxin transport assay. (A) Whole images of a soil-grown 6-wk-old wild-type plant (left) and its simulation at step 2,000 (right), where pink frames show the basal part, used for the polar auxin transport assay or its simulation. (B) An inverted stem segment whose apical end is being incubated with radiolabelled 1 µM IAA (left) and its simulation (right). In the polar auxin transport assay, 5 mm of the basal end (pink frame in B left) is excised after 6-h incubation to measure basipetally transported auxin. In its simulation, auxin concentration in the apical metamer (i) was set to be high (10) and constant over time, and initial auxin concentration in the basal metamer (j, shown in a pink frame) was assumed to be zero, because the concentration of 1 µM radiolabelled IAA is much higher than endogenous IAA concentrations, which typically range from pM to nM [62]. Based on the assumption that 6 wk are approximately equivalent to 2,000 steps in simulation, the incubation period of 6 h was simulated with 10 steps. Auxin concentration in the basal metamer at step 10 was calculated by using both PIN concentration values retrieved from whole plant simulation at step 2,000 and Equations 1 and 2 in Prusinkiewicz et al. (2009) [20], converted to a percentage of the wild-type simulation, and is shown as the polar auxin transport level in Table 1 and Figure 2.
(DOC)

## Abbreviations

2: 4-D, 2,4-Dichlorophenoxyacetic acid
PATS: polar auxin transport stream
PM: plasma membrane
s.e.m.: standard error of the mean
