# Supplementary material for: Strigolactone Can Promote or Inhibit Shoot Branching by Triggering Rapid Depletion of the Auxin Efflux Protein PIN1 from the Plasma Membrane
Source: PLoS Biol. 2013 Jan 29;11(1):e1001474. doi: 10.1371/journal.pbio.1001474 (PMC3558495; doi:10.1371/journal.pbio.1001474)
Supplement: Figure S1 — Auxin transport through inflorescence stem segments of pin1 mutants is strongly reduced. Auxin transport in pin1 mutant stem segments was assessed as previously described [19]. The mean amount of apically supplied radiolabelled auxin (counts per minute) transported to the basal end of stem segments is shown, ± the standard error or the mean, n = 20. These results are consistent with previous reports [4]. (DOC) [file pbio.1001474.s001.doc]

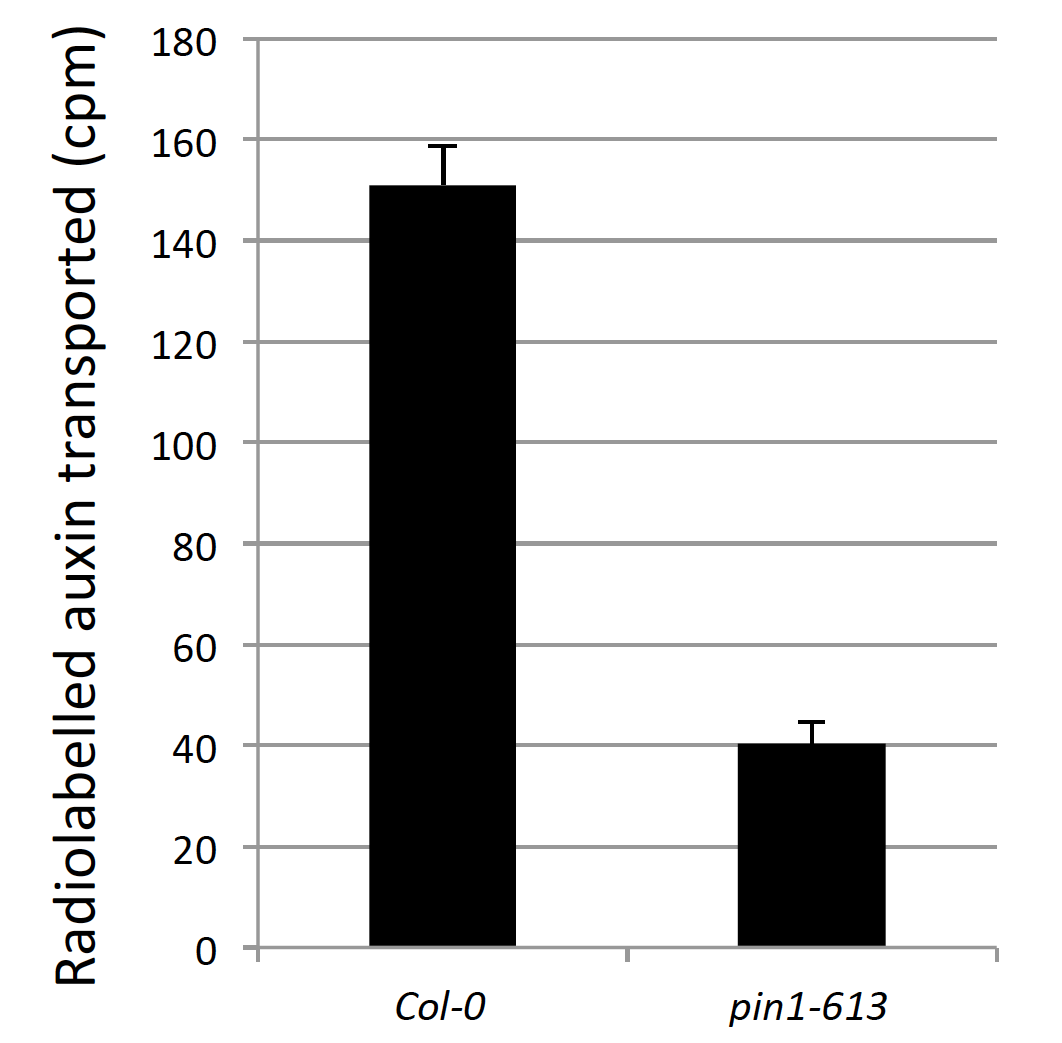


Supplementary Figure S1: Auxin transport through inflorescence stem segments of *pin1* mutants is strongly reduced.

Auxin transport in *pin1* mutant stem segments was assessed as previously described [19]. The mean amount of apically supplied radiolabelled auxin (counts per minute) transported to the basal end of stem segments is shown, +/- the standard error or the mean, n=20. These results are consistent with previous reports [4].
