# Supplementary material for: Strigolactone Can Promote or Inhibit Shoot Branching by Triggering Rapid Depletion of the Auxin Efflux Protein PIN1 from the Plasma Membrane
Source: PLoS Biol. 2013 Jan 29;11(1):e1001474. doi: 10.1371/journal.pbio.1001474 (PMC3558495; doi:10.1371/journal.pbio.1001474)
Supplement: Figure S2 — An overview of experimental and simulation designs of the auxin transport assay. (A) Whole images of a soil-grown 6-wk-old wild-type plant (left) and its simulation at step 2,000 (right), where pink frames show the basal part, used for the polar auxin transport assay or its simulation. (B) An inverted stem segment whose apical end is being incubated with radiolabelled 1 µM IAA (left) and its simulation (right). In the polar auxin transport assay, 5 mm of the basal end (pink frame in B left) is excised after 6-h incubation to measure basipetally transported auxin. In its simulation, auxin concentration in the apical metamer (i) was set to be high (10) and constant over time, and initial auxin concentration in the basal metamer (j, shown in a pink frame) was assumed to be zero, because the concentration of 1 µM radiolabelled IAA is much higher than endogenous IAA concentrations, which typically range from pM to nM [62]. Based on the assumption that 6 wk are approximately equivalent to 2,000 steps in simulation, the incubation period of 6 h was simulated with 10 steps. Auxin concentration in the basal metamer at step 10 was calculated by using both PIN concentration values retrieved from whole plant simulation at step 2,000 and Equations 1 and 2 in Prusinkiewicz et al. (2009) [20], converted to a percentage of the wild-type simulation, and is shown as the polar auxin transport level in Table 1 and Figure 2. (DOC) [file pbio.1001474.s002.doc]

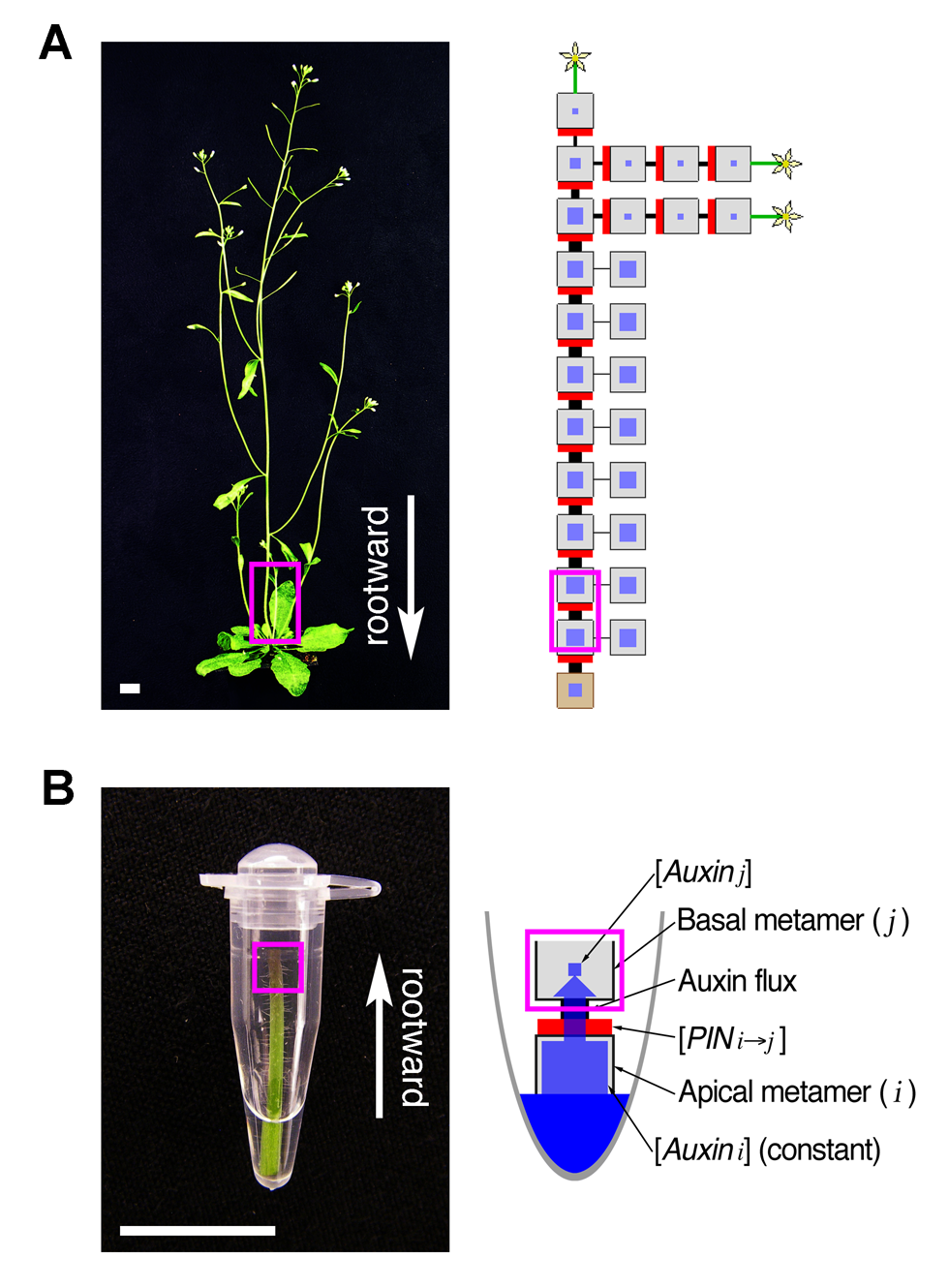


Supplementary Figure S2: An overview of experimental and simulation designs of auxin transport assay.

(A) Whole images of a soil-grown 6-week-old wild-type plant (left) and its simulation at step 2000 (right), where pink frames show the basal part, used for polar auxin transport assay or its simulation. (B) An inverted stem segment whose apical end is being incubated with radiolabelled 1 µM IAA (left), and its simulation (right). In polar auxin transport assay, 5 mm of the basal end (pink frame in B left) is excised after 6-hour–incubation to measure basipetally transported auxin. In its simulation, auxin concentration in the apical metamer (i) was set to be high (10) and constant over time, and initial auxin concentration in the basal metamer (j, shown in a pink frame) was assumed to be zero, because the concentration of 1 µM radiolabelled IAA is much higher than endogenous IAA concentrations, which typically range from pM to nM [62]. Based on the assumption that 6 weeks are approximately equivalent to 2000 steps in simulation, the incubation period of 6 hours was simulated with 10 steps. Auxin concentration in the basal metamer at step 10 was calculated by using both PIN concentration values retrieved from whole plant simulation at step 2000 and the equations (1) and (2) in Prusinkiewicz et al. (2009) [20], converted to the percentage to wild-type simulation, and is shown as the polar auxin transport level in Table 1 and Figure 2.
